# Porous Polyimide Membranes Prepared by Wet Phase Inversion for Use in Low Dielectric Applications

**DOI:** 10.3390/ijms14058698

**Published:** 2013-04-24

**Authors:** Soohyun Kim, Keon-Soo Jang, Hee-Dok Choi, Seung-Hoon Choi, Seong-Ji Kwon, Il-Doo Kim, Jung Ah Lim, Jae-Min Hong

**Affiliations:** 1 Energy Institute, Energy and Mineral Engineering, Pennsylvania State University, University Park, PA 16801, USA; E-Mail: syk5336@psu.edu; 2 Future Convergence Research Division, Korea Institute of Science and Technology, Seoul 136-791, South Korea; E-Mails: hdchoi@kist.re.kr (H.-D.C.); shchoi@kist.re.kr (S.-H.C.); sjkwon@kist.re.kr (S.-J.K.); ilkim@kist.re.kr (I.-D.K.); jalim@kist.re.kr (J.A.L.); 3 Department of Macromolecular Science and Engineering, Case Western Reserve University, Cleveland, OH 44106, USA; E-Mail: ksjang4444@gmail.com

**Keywords:** low K material, porous polyimide, poly amic acid, wet phase inversion

## Abstract

A wet phase inversion process of polyamic acid (PAA) allowed fabrication of a porous membrane of polyimide (PI) with the combination of a low dielectric constant (1.7) and reasonable mechanical properties (Tensile strain: 8.04%, toughness: 3.4 MJ/m^3^, tensile stress: 39.17 MPa, and young modulus: 1.13 GPa), with further thermal imidization process of PAA. PAA was simply synthesized from purified pyromellitic dianhydride (PMDA) and 4,4-oxydianiline (ODA) in two different reaction solvents such as γ-butyrolactone (GBL) and *N*-methyl-2-pyrrolidinone (NMP), which produce M_w_/PDI of 630,000/1.45 and 280,000/2.0, respectively. The porous PAA membrane was fabricated by the wet phase inversion process based on a solvent/non-solvent system via tailored composition between GBL and NMP. The porosity of PI, indicative of a low electric constant, decreased with increasing concentration of GBL, which was caused by sponge-like formation. However, due to interplay between the low electric constant (structural formation) and the mechanical properties, GBL was employed for further exploration, using toluene and acetone *vs.* DI-water as a coagulation media. Non-solvents influenced determination of the PAA membrane size and porosity. With this approach, insight into the interplay between dielectric properties and mechanical properties will inform a wide range of potential low-k material applications.

## 1. Introduction

Aromatic polyimides (PIs) demonstrate good material characterization such as high thermal stability and good mechanical strength [1]. They have been also widely used as insulating layers and buffer coatings in electronic packaging, indicating a good candidate of low-k dielectric materials [1]. The low dielectric constant material can solve the electric drawbacks such as interconnection or signal delay for high performance nano-electric devices [2]. Unfortunately, the dielectric value of raw PI materials is approximately 3.4 at 1 kHz-1 MHz [3]. Therefore, chemical modifications of PI have been routinely introduced by copolymerizing various polyimide precursors in an effort to tune the material for the desired electrical properties. For example, hydrogen atoms may be substituted with –F or –CF_3_ groups because fluorination tends to decrease a material’s dielectric constant [4]. The dielectric constants of most fluorinated polyimides are still high with the range of 2.6–3.0. Introducing micro-porous structures into a polymer matrix is another attractive approach to decreasing the dielectric constant by providing an architecture containing embedded air pockets (with a dielectric constant of 1).

Porous polyimides have been prepared using several methods, including blowing agents [5], thermally labile urethane prepolymers [6], ceramic components [7], and porogens [8]. Such methods require additives to generate pores in a polymer matrix, whereas phase inversion processes can provide asymmetric porous polymer films from a homogeneous polymer solution. Wet phase inversion processes are usually utilized by immersing a thin cast polymer solution into a non-solvent [9].

Among the polyimides synthesized from combining various precursors, the rigid aromatic poly(4,4′-oxydiphenylenepyromellitimide) has been popular for use in commercial films (Kapton by Dupont). However, the insolubility of this polymer limits its wet processability for use in applications. This polyimide cannot be used to prepare porous membranes via wet phase inversion processes. Porous polyimide membranes are prepared via the wet phase inversion and the dry phase inversion. The wet phase inversion for PI requires soluble and less rigid polyimides, which gives limitations.

To overcome this drawback, Echigo *et al.* reported that porous poly(4,4′-oxydiphenylenepyromellitimide) can be prepared using the dry phase inversion from pyromellitic dianhydride (PMDA) and 4,4-oxidianiline (ODA) [10]. PMDA and ODA were polymerized in tetrahydrofuran (THF)/methanol (MeOH) to form a prepolymer of poly(4,4′-oxydiphenylene pyromellitimic acid (PAA). The non-solvent, H_2_O for this dry phase inversion system was added to the polymer solution to induce the phase inversion. A film cast from the resulting solution was dried and thermally cured to convert the PAA to PI via imidization. During the drying process, a microporous structure was developed. The required cooling bath for this method limits choice of solvent for those with a lower boiling point than H_2_O.

Due to the drawbacks in these previous explorations, we designed a novel approach to the fabrication of porous PAA. PAA was prepared in a common solvent such as as γ-butyrolactone (GBL) and *N*-methyl-2-pyrrolidinone (NMP), and the resulting PAA solution was submitted to a wet phase inversion process without solidification to form a porous membrane. The PAA membrane consisted of an asymmetric porous structure that was transformed to PI via thermal imidization. Various solvent systems based on NMP and GBL were selected for PAA polymerization. We investigated the effect of the different solvent composition on the porous PAA membrane morphology. The effects of a coagulation bath composed of the non-solvents such as acetone, toluene, and DI-water were examined. The dielectric and mechanical properties of the material were characterized with the aim of using this membrane as a low-k material in applications.

## 2. Results and Discussion

### 2.1. Polyimidazation of Porous Polyamic Acid

The PAA solutions were synthesized from PMDA/ODA in each of the two different solvents (NMP and GBL) After polymerization of PAA, the two different molecular weight PAAs in NMP and GBL, respectively, were mixed in different ratios (100/0, 70/30, 50/50, 30/70, 10/90, and 0/100) based on the volume fraction at room temperature. During the polymerization, the reactivity depended on the type of PDMA added, such as a lump or a powder. The lump PMDA added to the ODA solution decelerates the polymerization as the PMDA is dissolved, thereby reaching a higher molecular weight than attained from the powder PMDA. Thus, polymerization using the lump PMDA produces desired high molecular weight polymers. The polymer solution obtained from the PMDA powder had a low viscosity and failed to form asymmetric membranes via the wet phase inversion.

As the ratio of GBL/NMP increased from zero (pure NMP), the solution viscosity increased. In pure GBL, the viscosity of the polymer solution from a lump of PMDA increased dramatically, and the reaction ceased due to the high viscosity before the reaction reached completion. Therefore, polymerization in pure GBL using the powdered PMDA was more extensive.

The molecular weight/polydispersity index (PDI) of the PAA synthesized in NMP and GBL were 280,000/2.0, and 630,000/1.45, respectively. PAA in GBL (0/100) exhibited a high molecular weight with a narrower molecular distribution than those in the case of NMP (100/0). The solvent affected the molecular weight of the PAA by altering the reactivity [11]. The formed ortho-dicarboxylic group during the reaction may be highly stabilized due to the acid-base interaction with the dipolar aprotic solvent (NMP) and thus exists as one of the end groups of the PAA, thus effectively lowering the molecular weight [12]. Therefore, the reactivity between PMDA and ODA in GBL toward PAA was better than in NMP, which resulted in high molecular weight PAA.

**Figure 1:**
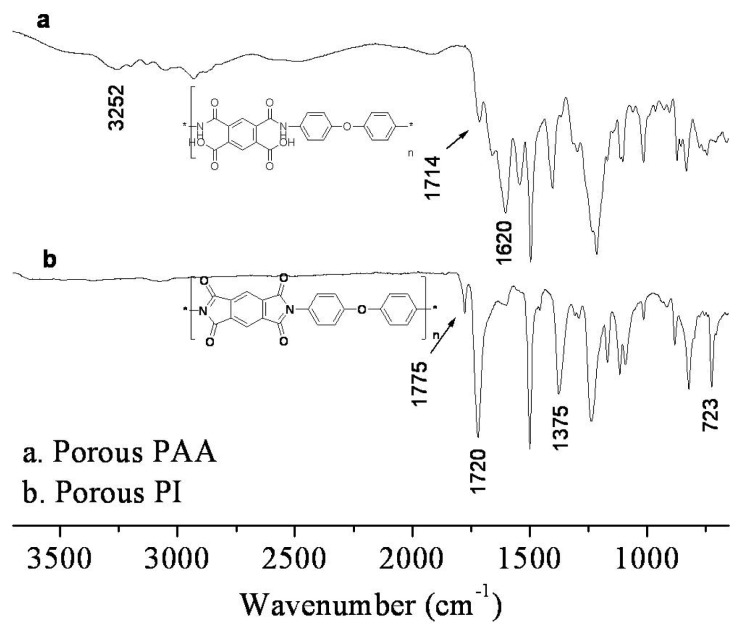
ATR FT-IR spectra of porous membrane of (**a**) polyamic acid (PAA) (solid) and (**b**) polyimide (PI) (dot) by thermal imidization.

PAA solutions with different solvent compositions were cast onto a glass plate, allowing 2–3 s, and coagulated in a DI-water bath. Because the solvents transition permeative to water from the coagulation bath, the transparent PAA solution was transformed into an opaque film. All PAA solutions formed asymmetric membranes with macro-voids or porous structures. The obtained films were dried and thermally cured to convert to PI. To confirm the imidization, macro-void membranes formed from the NMP (100/0) solution were characterized by ATR FT-IR before and after thermal curing. The PAA-to-PI transition was monitored by ATR FT-IR, as shown in the spectra of Figure 1.

The broad peaks around 3252 cm^−1^ and 1620 cm^−1^ indicated the characteristic PAA N-H and amide C=O stretching modes, respectively. The peaks disappeared in the spectra of the membrane after thermal curing, and peaks at 1775 cm^−1^ (C=O asymmetric stretching), 1720 cm^−1^ (C=O symmetric stretching), 1375 cm^−1^ (CN stretching), and 723 cm^−1^ (C=O bending), which is primary indication of PI absorption bands, transition bigger with no characteristic PAA peaks. The ATR FT-IR spectra indicated that the porous PAA membranes were successfully converted to PI [8].

### 2.2. Effect of the Different Solvent Ratio to Form the Membrane

**Figure 2:**
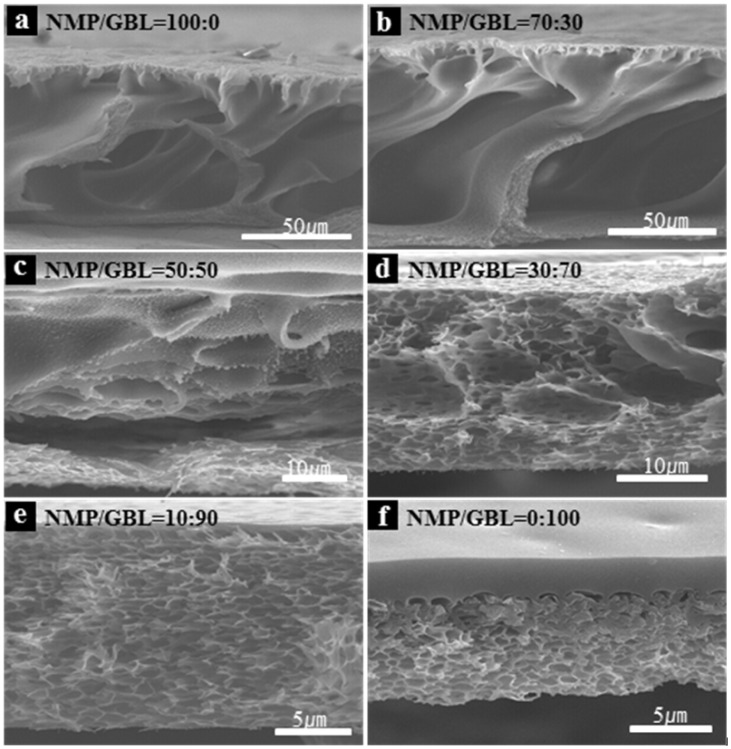
Cross-sectional SEM images of polyimide membranes as a function of the *N*-methyl-2-pyrrolidinone (NMP)/γ-butyrolactone (GBL) ratio in DI-water.

Figure 2 shows cross-sectional SEM images of the porous membranes prepared from different solvent compositions using DI-water as a coagulation bath. The PAA/NMP membrane formed macro-voids of 60 μm in size, which may lead to a low dielectric constant. Unfortunately, the macro-void membrane showed fragility for further electrical and mechanical tests. The void size depended on the composition of the solvent. As the GBL content increased from 0 to 100%, the void size decreased, which also indicates a transition from a macrovoid structure to a sponge-like structure.

The sponge-like and the macrovoid structures should be comprehended in terms of both enlarged miscibility gap thermodynamically and increased polymer solution viscosity kinetically. The solubility parameter and heat of mixing allow the determination of miscibility between the polymer and the solvent, and two different solvents, respectively. Based on Hildebrand solubility parameter data, the solubility parameter of NMP (23.1 MPa^1/2^) is closer to that of PAA (20.8 MPa^1/2^) than GBL (25.2 MPa^1/2^) is. According to the literature [13], mixing GBL with water was found to be endothermic in all compositions while NMP/water was exothermic. As NMP is mixed with water more favorably than GBL is, the solution with NMP undergoes fast phase-separation and represent the macrovoid structure. The spongelike structure in the GBL system may be the result of low interactions of GBL/water [14] In general, the endothermic process accompanies an increase of entropy, and thus, GBL produces more pores. Based on the solubility parameter gap between PAA and the solvent, and heat of mixing between the solvent and the non-solvent, indicative of miscibility, the characteristic thermodynamic features of the PAA/GBL/water system might inhibit phase separation from proceeding further, undergoing insufficient liquid-liquid phase separation that induces the spongelike structure [15]. In addition, higher molecular weight of PAA in GBL leads to the enlarged polymer solution viscosity, which then causes the suspension of the macrovoid structure [15].

### 2.3. Effects of the Coagulation Medium on the Porous Morphology

The porous morphology prepared during the wet phase inversion process can be adjusted by introducing a non-solvent to the coagulating bath [16]. We investigated various membrane morphologies prepared using PAA/NMP and PAA/GBL solutions, coagulated in other non-solvents: acetone, toluene, and DI-water.

**Figure 3:**
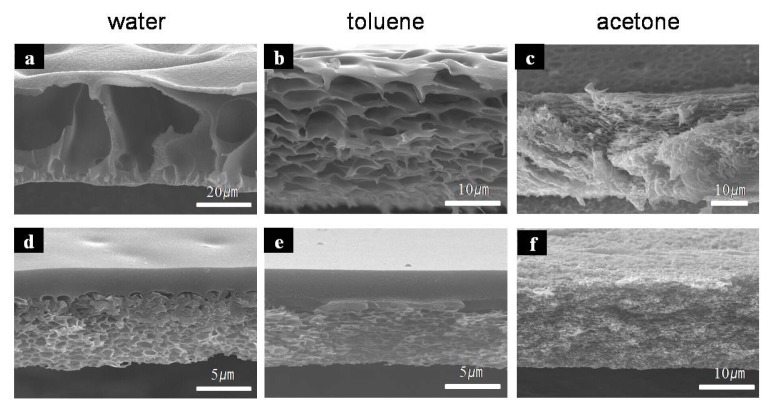
Cross-section SEM images of polyimide membrane in the different coagulation baths of DI-water, toluene, acetone; (**a**–**c**) of PAA/NMP; (**d**–**f**) of PAA/GBL.

Figure 3 shows cross-sectional images of porous membranes formed from two kinds of PAA solutions in different coagulation media via the phase inversion process. In both polymer solutions, the pore size of the membrane decreased with incorporation of DI-water, toluene, and acetone, respectively. In the case of acetone, the morphologies of both polymers were sponge-like (Figure 3c,f) and the top skin layers were more porous than those of other cases. The porosity was caused by the low affinity between the solvent and the non-solvent. Slow mixing of the solvent and non-solvent can initiate many nuclei simultaneously to form sponge-like structures with plenty of small pores [7]. NMP and GBL, therefore, had a high affinity toward water and a low affinity toward acetone.

The morphologies of membranes prepared from the low molecular weight PAA/NMP system varied more significantly among the coagulation baths than did those prepared from the high molecular weight PAA/GBL system. The pore sizes in the PAA/NMP membranes gradually decreased as water was replaced by acetone in the coagulation baths. On the other hand, the PAA/GBL/water membrane was similar to the PAA/GBL/toluene membrane and consisted of a thick dense skin layer and a porous sub-layer. The dense skin layer increased the strength modulus relative to the porous skin layer. Therefore, morphologies with porous structures below a dense skin layer may potentially be useful in low-k applications.

### 2.4. Effects of the Coagulation Medium on the Porous Morphology

Mechanical stress in electronic devices must be minimized to extend device lifetimes. Mechanical stress can cause chip package deformation, adhesion loss between components, or cracking of the package material. Therefore, porous PI films require the combination of a porous structure and strong mechanical strength. In general, the dense PI films composed of PMDA-ODA represent excellent mechanical properties, and high tensile strengths and moduli.

The mechanical properties of a raw PI film (non-porous) and the porous PI films fabricated by the phase inversion process using the GBL solvent in conjunction with the coagulation baths (acetone or toluene) were measured by tensile tests., as shown in Table 1. Nonporous PI films were prepared by direct thermal imidization without the phase inversion process. The films formed in a DI-water bath were not measured due to the film fragility. The nonporous PI films, with approximately 10 μm in thickness, were characterized by UTM. The average film thickness obtained from acetone and toluene (Table 1) was 20–30 μm. Comparing to acetone, the mean strength, tensile strength load, and modulus of the porous films formed in toluene were enhanced from 1.48%, 3.93 MPa, and 0.27 GPa to 8.04%, 39.17 MPa, and 1.13 GPa, respectively. The mechanical strength and the modulus in the case of toluene were somewhat lower than those of the raw PI film, but 10 and 4 times higher than those of the porous film formed in acetone, respectively due to the presence of a dense top layer. Interestingly, the toughness and the elongation at break using toluene (8.04% and 3.4 MJ/m^3^) as a coagulation bath was 3 and 2 times higher than that of the raw PI (2.68% and 1.8 MJ/m^3^) without the wet inversion process.

### 2.5. Measurement of Dielectric Constant

**Figure 4:**
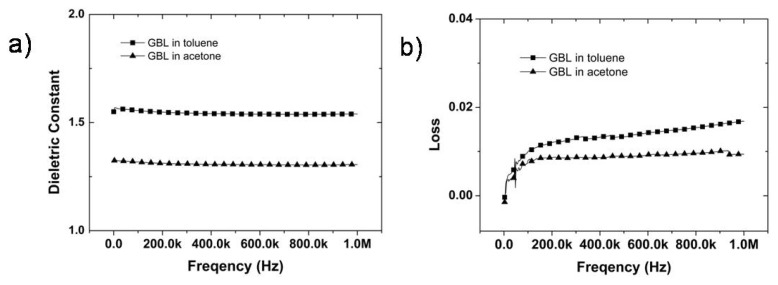
Dielectric properties of porous polyimides formed in GBL solutions; (**a**) dielectric constant; (**b**) dielectric loss.

The dielectric constants and losses of porous PI films obtained from GBL solutions were measured between 1 kHz and 1 MHz using an impedance analyzer, as shown in Figure 4. The average dielectric constants and dielectric loss of the porous PI films at 1 MHz were 1.60 and 0.015 for GBL/toluene and 1.32 and 0.008 for GBL/acetone, respectively (Figure 4a,b). The dielectric constant of the GBL/acetone system was approximately 20% lower than that of the GBL/toluene system due to the sponge-like morphology, which results in low mechanical properties. The porous membrane of the GBL/acetone system displayed higher porosity with more air voids than that of the GBL/toluene system. However, due to the interplay between the mechanical properties and the electric properties, it is asserted that the GBL/toluene system is the most suitable system among any other case.

Incorporation of micro-porous structures into a polymer matrix induces significant decrease in the dielectric constant and low dielectric loss by providing an architecture containing embedded air pockets. Because the structure was quiet stable at high frequency, the dielectric loss demonstrated markedly low value. The major problem of PI film with porous structures was poor mechanical strength even though it has low dielectric constant and markedly low dielectric loss of materials. This problem could be solved by asymmetric structure between dense skin layer and air-capped sponge like structure. Therefore, this approach may potentially be very useful in further low-k applications.

## 3. Experimental Section

Pyromellitic dianhydride (PMDA) and 4,4-oxidianiline (ODA) are commercial products purchased from Aldrich. PMDA and ODA were purified by vacuum sublimation, which resulted in formation of a lump material. *N*-Methyl-2-pyrrolidone (NMP, Aldrich Co.) and γ-butyrolactone (GBL, Acros) were used to form a casting solvent without further purification.

Synthesis of polyamic acid: The purified ODA was dissolved in the NMP/GBL co-solvent to a concentration of 5 w/w% under dry nitrogen. PMDA was added to NMP then dissolved in the NMP/GBL co-solvent. PMDA powder was dissolved in GBL, after having precipitated from the dissolved lump material. The two solutions were stirred at room temperature for 8–12 h (co-solvent solution) and 4–6 h (GBL solution).

Membrane preparation: Two different molecular weight PAAs were polymerized by using two different solvents; 100% GBL and 100% NMP, respectively to avoid any effect from reaction mixture of two solvents during polymerization. After each polymerization, we simply added two different compositions of PAA(*M*_w_ = 280,000)/NMP and PAA(*M*_w_ = 630,000)/GBL via tailored composition; 100:0, 70:30, 50:50, 30:70, 10:90, and 0:100 based on the volume fraction. A viscous 10 w/w% PAA solution was cast onto a glass plate to a thickness of 120 μm. The evaporation time was few seconds (2–3 s) before immersing the PAA solution into a coagulation bath at room temperature. De-ionized water (18.2 MΩ, obtained from a Millipore-Q Elix 10), acetone, and toluene were used as a coagulation bath. The resulting membranes were incubated in the solution for 2 h then dried under vacuum at room temperature. The membranes were cured at 100 °C, 200 °C, and 300 °C for each 1 h.

The molecular weight of the PAA was determined by gel permeation chromatography (GPC), using dimethylacetamide (DMAc) as the eluent. The chemical structures were investigated by ATR FT-IR spectroscopy (Perkin Elmer). The tensile strengths of the PI were measured using a Universal Test Machine (UTM) with a 5 mm width by 6 cm length film. Electrical measurements were conducted using 100 nm thick Al electrodes (area = 0.43 cm^2^, 0.36 cm^2^) deposited through a shadow mask onto the PI membranes. The dielectric properties were measured between 1 kHz and 1 MHz using an impedance analyzer (HP 4194A).

Scanning electron microscopy (SEM) (S-4100, HITACHI) was employed to observe the development of the microstructure. The porous membrane was coated by Pt/Pd alloy metal on the holder to see SEM images. For SEM cross-section of membrane, the porous membrane was cut in the liquid nitrogen with tweezers to preserve the original formation of samples. By the opposite force of membrane, the membrane was successfully broken without damages due to liquid nitrogen. The broken membrane was blown by a small air-brush to remove any artifacts completely. Then, the cross-sections of membrane were observed on the side-view holder by SEM.

## 4. Conclusions

We successfully fabricated porous PI asymmetric membranes via thermal imidization of a porous PAA membrane after the wet inversion process of PAA using two different solvents; NMP and GBL. The PAA solution was prepared from PMDA and ODA. The PAA pre-polymer with two different molecular weights was synthesized. The resulting solutions with various ratios between NMP and GBL were immersed in a coagulation bath and formed porous membranes with various morphologies. A liquid–liquid de-mixing process using two solvents, NMP and GBL, altered the membrane structure from a macro-porous structure to a sponge-like structure as a function of the NMP/GBL ratio. A variety of porous membrane structures with macro-voids or dense skin layers were obtained using different coagulation media. A PI film with porous structures below a dense skin layer fabricated from a GBL/toluene system exhibited good mechanical properties and a low dielectric constant value. The toughness from a porous PI membrane examined in this study was higher than the non-porous PI membrane. The dielectric properties of the porous PI membranes showed a remarkable low dielectric loss of <0.015 at 1 MHz while retaining a dielectric constant of <1.7. This material may potentially be useful in low-k applications.

## Figures and Tables

**Table 1:** Tensile strength properties of porous polyimide film and nonporous.

Coagulation media	Tensile strain at maximum load (%)	Tensile stress at maximum load (MPa)	Young modulus (GPa)	Toughness (MJ/m^3^)
Acetone	1.48	3.93	0.27	0.1
Toluene	8.04	39.17	1.13	3.4
nonporous	2.68	48.67	2.64	1.8
